# Bacterial Communities Associated with the Surfaces of Fresh Fruits and Vegetables

**DOI:** 10.1371/journal.pone.0059310

**Published:** 2013-03-27

**Authors:** Jonathan W. Leff, Noah Fierer

**Affiliations:** 1 Cooperative Institute for Research in Environmental Sciences, University of Colorado, Boulder, Colorado, United States of America; 2 Department of Ecology and Evolutionary Biology, University of Colorado, Boulder, Colorado, United States of America; Graz University of Technology (TU Graz), Austria

## Abstract

Fresh fruits and vegetables can harbor large and diverse populations of bacteria. However, most of the work on produce-associated bacteria has focused on a relatively small number of pathogenic bacteria and, as a result, we know far less about the overall diversity and composition of those bacterial communities found on produce and how the structure of these communities varies across produce types. Moreover, we lack a comprehensive view of the potential effects of differing farming practices on the bacterial communities to which consumers are exposed. We addressed these knowledge gaps by assessing bacterial community structure on conventional and organic analogs of eleven store-bought produce types using a culture-independent approach, 16 S rRNA gene pyrosequencing. Our results demonstrated that the fruits and vegetables harbored diverse bacterial communities, and the communities on each produce type were significantly distinct from one another. However, certain produce types (i.e., sprouts, spinach, lettuce, tomatoes, peppers, and strawberries) tended to share more similar communities as they all had high relative abundances of taxa belonging to the family Enterobacteriaceae when compared to the other produce types (i.e., apples, peaches, grapes, and mushrooms) which were dominated by taxa belonging to the Actinobacteria, Bacteroidetes, Firmicutes, and Proteobacteria phyla. Although potentially driven by factors other than farming practice, we also observed significant differences in community composition between conventional and organic analogs within produce types. These differences were often attributable to distinctions in the relative abundances of Enterobacteriaceae taxa, which were generally less abundant in organically-grown produce. Taken together, our results suggest that humans are exposed to substantially different bacteria depending on the types of fresh produce they consume with differences between conventionally and organically farmed varieties contributing to this variation.

## Introduction

Fresh produce, including apples, grapes, lettuce, peaches, peppers, spinach, sprouts, and tomatoes, are known to harbor large bacterial populations [1]–[7], but we are only just beginning to explore the diversity of these produce-associated communities. We do know that important human pathogens can be associated with produce (e.g., *L. monocytogenes*, *E. coli*, *Salmonella*), and since fresh produce is often consumed raw, such pathogens can cause widespread disease outbreaks [8]–[11]. In addition to directly causing disease, those microbes found in produce may have other, less direct, impacts on human health. Exposure to non-pathogenic microbes associated with plants may influence the development of allergies [12], and the consumption of raw produce may represent an important means by which new lineages of commensal bacteria are introduced into the human gastrointestinal system. More generally, produce-associated microbes can have important effects on the rates of food spoilage [13], and many of the microbes found on kitchen surfaces appear to come from produce sources [14].

Previous work investigating microbial communities on fresh produce has generally focused on culturable pathogenic bacteria and fungi (*sensu* [9]) with only a few recent studies having assessed the composition of produce-associated microbial communities using culture-independent techniques. From this previous work, a few key patterns emerge: (1) Different produce types and cultivars can harbor different abundances of specific bacterial groups [9], (2) farming and storage conditions may influence the composition and abundances of microbial communities found on produce [3], [5], [15]–[18], and (3) non-pathogenic microbes may interact with and inhibit microbial pathogens found on produce surfaces [7], [9], [19]–[21]. Despite this body of work, we still have a limited understanding of the diversity of produce-associated microbial communities, the factors that influence the composition of these communities, and the distributions of individual taxa across produce types (particularly those taxa that are difficult to culture).

We expected the overall composition of microbial communities to vary across produce types for a variety of reasons. First, we know from previous work on tree leaf surfaces that different plant lineages are likely to harbor very distinct bacterial communities [22], [23]. Moreover, we know that a range of environmental factors which can shape microbial community composition, including pH and moisture availability, can vary across produce types [6], [13], [24]. Likewise, differences in growing conditions, transport procedures, and storage conditions could influence the diversity and composition of produce-associated microbial communities. For example, we would expect produce grown closer to the ground to have higher relative abundances of soil microbial taxa and produce stored at cold temperatures for longer periods of time may harbor greater abundances of cold-tolerant bacteria [6], [15], [16], [25].

Farming practices may also have an important, but understudied, influence on the composition of produce-associated microbial communities. Consumers in developed nations are commonly exposed to differences in farming practices through their choice between organic and conventionally farmed produce items. Organic farming practices can differ from conventional farming practices in a variety of ways, including the types of fertilizer and pesticides that are used, and these differences have the potential to impact microbial community structure on produce surfaces [4], [17], [18], [26]. However, we do not know if these potential effects of farming practices on produce-associated microbial communities are evident across a wide range of produce types and whether such effects persist up until the point that produce is purchased and consumed.

The objective of this study was to characterize the bacterial communities on the surfaces of multiple types of fruits and vegetables at the point of sale. We focused on those produce types that are frequently consumed raw, as we are likely exposed to far more live bacteria when we consume raw foods compared to cooked foods. Specifically, we addressed two fundamental questions: (1) How does bacterial community structure differ among produce types? and (2) Do differences in farming practices, such as those used on conventional and organic farms, have the potential to influence the composition of bacterial communities on the surfaces of produce items as experienced by end consumers? Because we know that culture-based techniques do not adequately capture a large portion of bacterial diversity on produce [27], we addressed these questions using high-throughput pyrosequencing analysis of the 16 S rRNA gene found in bacterial DNA extracted from the surfaces of the produce items.

## Materials and Methods

### Sample Collection

Fresh produce items were purchased from three differently-branded grocery stores in Boulder, CO, USA. These items consisted of eleven produce varieties, and for nine of these, both organic and conventional-labeled versions were obtained. Produce varieties and numbers of replicates are described in Table 1. In the USA, organically farmed produce differs from conventionally farmed produce in that synthetic pesticides and fertilizers, ionizing radiation, and sewage are generally not allowed in its production (http://www.usda.gov/). We acknowledge that differences observed between conventional and organic-labeled produce could be attributable to a number of factors that are not necessarily reflective of the differences in farming practices represented by the label. These include potential differences in farm location, transport, storage conditions, and storage time. However, these factors are difficult to control and thus our goal in this study was to assess the *potential* for broad-scale differences in farming practices to affect bacterial communities on produce items available to end consumers.

**Table 1:** Produce varieties and sample numbers.

		Sample replicates (purchased)		Sample replicates (after rarefaction)	
Produce variety	Bacteria sampling method	Conventional	Organic	Conventional	Organic
Apple (*Malus domestica* “Granny Smith”)	swab	12	12	9	8
Grapes (*Vitis vinifera*)	rinse	12	8	10	8
Lettuce (*Lactuca sativa* var. *longifolia*)	rinse	12	8	10	7
Mushrooms (*Agaricus bisporus*)	swab	12	4	12	4
Peach (*Prunus persica*)	swab	12	8	12	6
Pepper (*Capsicum annuum* “bell”)	swab	12	12	10	12
Spinach (*Spinacia oleracea*)	rinse	11	12	8	12
Strawberries (*Fragaria × ananassa*)	swab	12	12	12	12
Tomato (*Solanum lycopersicum*)	swab	12	12	12	11
Alfalfa sprouts (*Medicago sativa*)	rinse	12		10	
Mung bean sprouts (*Vigna radiate*)	rinse	8		7	

Lettuce and spinach samples were pre-rinsed and sold pre-packaged, other produce items were collected either in store packaging (grapes, lettuce, mushrooms, spinach, sprouts, and strawberries) or sterile plastic bags (apples, peaches, peppers, and tomatoes). Replicate samples were collected from discrete packages (of the same brand) at each store when sold pre-packaged and replicate samples of other produce types were collected from discrete fruits. Bacterial samples were collected from each produce sample within a store on the same day, and each of the three stores were sampled within a single week. Bacterial samples were taken from produce samples using either sterile cotton swabs (following the procedure described in [14]) or by using a sterile water rinse to reduce the collection of chloroplasts (Table 1). For the rinsing procedure, water and produce samples were added to sterile plastic bags, and gently shaken for 5 min. Bacteria in the rinse water were collected onto 0.2 µm filters (Corning, Inc., Tewksbury, MA, USA) by vacuum filtration. Swabs and filters were stored at -20**°**C for less than 2 weeks prior to molecular analysis. DNA was extracted from swabs and filters using the PowerSoil-htp kit (Mo Bio Laboratories, Inc., Carlsbad, CA, USA) using modifications described previously [28].

### Determination of Bacterial Community Composition and Diversity

16 S rRNA gene sequences were analyzed via barcoded pyrosequencing to quantify the diversity and community composition of the bacterial communities associated with each of the 215 produce samples collected. They were amplified and sequenced from the extracted genomic DNA using a procedure described in [23]. Briefly, sequences were PCR amplified in triplicate using a primer pair (799 f/1115 r) which does not amplify chloroplast DNA [23], [29]. The reverse primer contained a 12-bp barcode sequence unique to each sample. The triplicate reactions were combined, DNA concentrations were measured, and equal quantities of DNA from each sample were combined together. The pooled DNA sample was cleaned using the UltraClean PCR Clean-Up Kit (Mo Bio Laboratories, Inc., Carlsbad, CA, USA) and sequenced at the Engencore facility at the University of South Carolina on the Roche 454 sequencing platform.

The 16 S rRNA gene sequences were processed using the QIIME v. 1.4.0 pipeline [30] to determine the diversity and composition of the produce-associated bacterial communities. Default parameters were used except that only sequences between 240 and 400 bp with both primers removed were retained for downstream analyses, and taxonomic identities were assigned to operational taxonomic units (OTUs) using the RDP classifier [31] trained on the Greengenes microbial 16 S rRNA gene sequence dataset (February 4, 2011 revision; greengenes.lbl.gov), clustered at a 97% similarity threshold. Because we obtained a variable number of sequences per sample (from only a few sequences to >4,000), the sequence data were rarefied at 200 sequences per sample to account for this variation. The rarefaction resulted in some samples being lost prior to further analysis, and information on the numbers of samples included in downstream analyses is provided in Table 1. At 200 sequences per sample, we were not able to survey the full extent of bacterial diversity in each sample, but previous work demonstrates that this depth of sampling is sufficient for accurate assessments of alpha and beta diversity patterns on both leaf surfaces [23] and in other microbial habitats [32]. Amplicon sequences were deposited in the public EMBL-EBI database (http://www.ebi.ac.uk/) and may be accessed using the accession number, ERP002018.

### Statistical Analyses

To assess differences in microbial community composition across the produce items (beta diversity), we calculated both phylogenetic metrics (weighted and unweighted UniFrac distances, [33], [34]) and a taxonomic metric (Bray-curtis dissimilarities calculated from log-transformed OTU abundances). Differences in overall bacterial community composition among the produce types and between farming practice type (organic versus conventional) were assessed using a permutational multivariate ANOVA test (PERMANOVA) with produce type and farming practice as fixed factors and the grocery store brand as a random factor. PERMANOVA tests were also used to test for the effects of farming practice on bacterial community composition within individual produce types. Significant differences in taxonomic richness were assessed across produce types using the nonparametric Kruskal-Wallis test and between conventional and organic labeled produce items using a t-test. Significant differences in the relative abundances of individual bacterial taxa across produce types or factor levels were determined using ANOVA and the false discovery rate (FDR) correction. T-tests were used when comparing the relative abundances of individual taxa between conventional and organic analogs. All multivariate analyses were performed using PRIMER 6 [35], and univariate analyses were performed using R [36].

## Results

### Differences in Bacterial Community Diversity and Composition Across Produce Types

**Figure 1:**
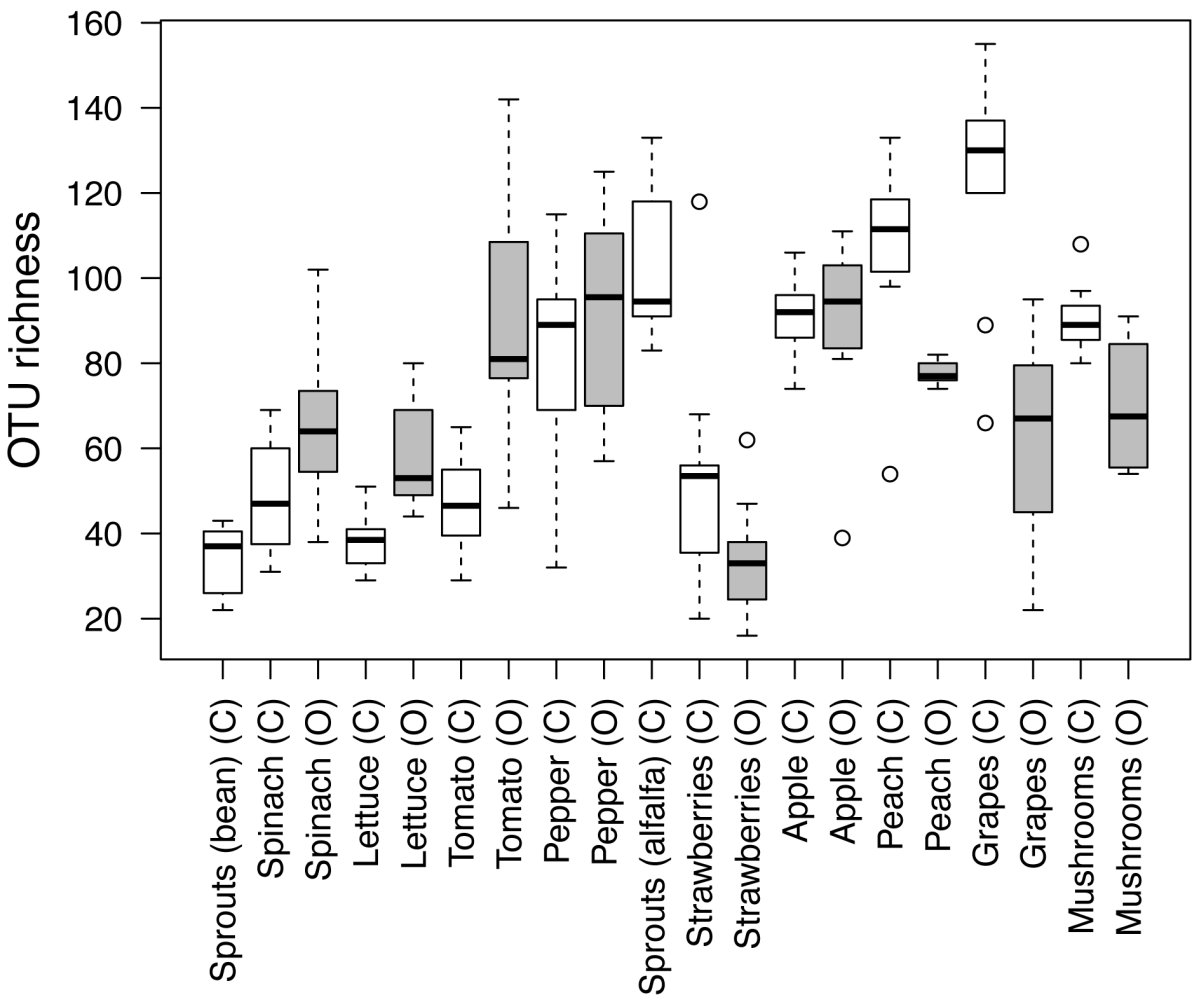
Boxplot of taxon richness for each produce type and conventional (C) and organic (O) equivalents. Samples were rarefied at 200 sequences per sample. Circles represent outliers.

**Figure 2:**
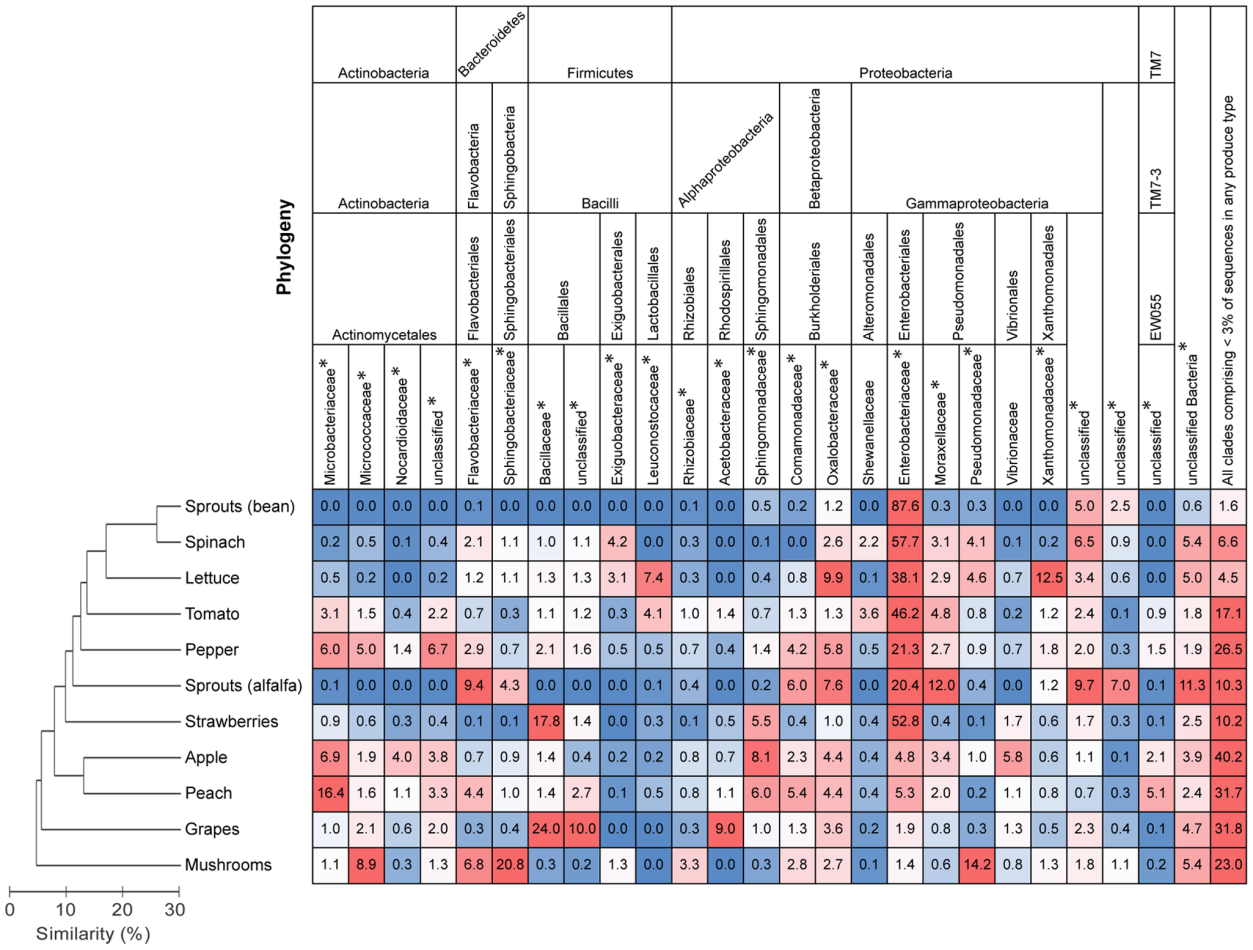
Relationships between bacterial communities on each produce type and relative abundances of bacterial families. The dendrogram is based on mean Bray-Curtis dissimilarities and shows differences among produce types in the overall composition of the bacterial communities. The heatmap shows mean relative abundances (%) of bacterial families on produce types. Only families and unclassified groupings representing at least three percent on any produce type are represented.

Although variable, taxonomic richness levels differed among the eleven produce types (*P*<0.001) with richness being highest on peaches, alfalfa sprouts, apples, peppers, and mushrooms and lowest on bean sprouts and strawberries (Fig. 1). Bacterial communities were highly diverse regardless of the produce type with between 17 and 161 families being represented on the surfaces of each produce type. However, the majority of these families were rare; on average, only 3 to 13 families were represented by at least two sequences per produce type. In some cases, OTUs assigned to a single bacterial family were dominant. For example, 88, 58, and 53% of OTUs on bean sprouts, spinach, and strawberries were assigned to the family Enterobacteriaceae, respectively. In contrast, the communities on apples were relatively even with no single family representing more than 8% of the sequences (Fig. 2).

Across the produce types, bacterial communities also differed with respect to their taxonomic structure, and produce type had a far larger influence on the observed variation in bacterial community composition than farming practice or store brand (Table 2). Furthermore, pairwise tests revealed that the community composition on the surface of each produce type differed significantly from one another (*P* = 0.001 in all cases; Fig. S1). Still, certain produce types shared more similar community structure than others. On average, tree fruits (apples and peaches) tended to share communities that were more similar in composition than they were to those on other produce types, and produce typically grown closer to the soil surface (spinach, lettuce, tomatoes, and peppers) shared communities relatively similar in composition. Surface bacterial communities on grapes and mushrooms were each strongly dissimilar from the other produce types studied (Fig. 2).

**Table 2:** PERMANOVA results of main factors.

Factor (type)	Diversity metric*	Pseudo-F	*P*	Component of variation
Produce type (Fixed)				
	Bray-Curtis	5.91	0.001	931
	Unweighted UniFrac	4.19	0.001	5.4×10^−2^
	Weighted UniFrac	19.0	0.001	1.4×10^−2^
Farming practice label (Fixed)				
	Bray-Curtis	2.96	0.001	78
	Unweighted UniFrac	2.16	0.001	4.1×10^−3^
	Weighted UniFrac	7.32	0.001	1.0×10^−3^
Store (Random)				
	Bray-Curtis	1.70	0.001	54
	Unweighted UniFrac	1.53	0.001	3.7×10^−3^
	Weighted UniFrac	2.48	0.001	4.6×10^−4^

* Bray-curtis dissimilarities were log transformed.

**Figure 3:**
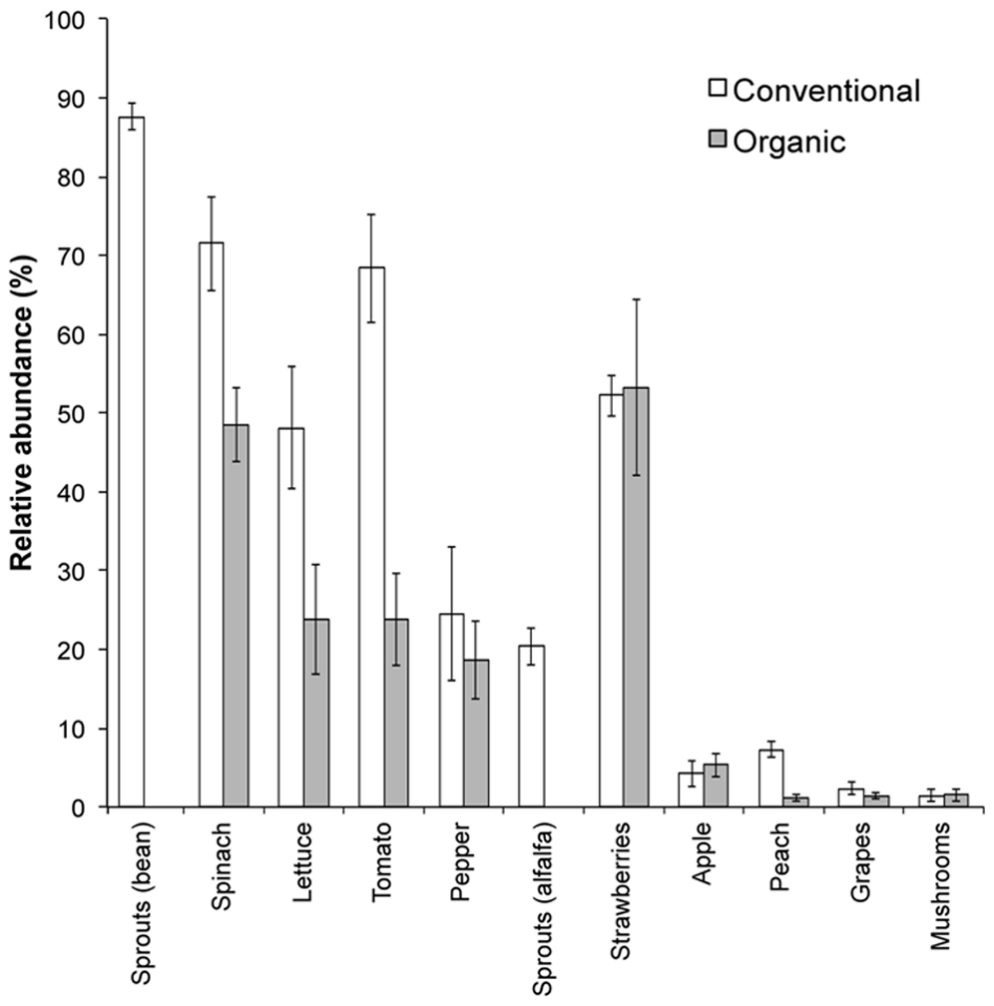
Mean relative abundances (±1 S.E.M) of bacteria belonging to the family Enterobacteriaceae. Each produce type and conventional and organic-labeled equivalents are shown. No organic-labeled equivalents were sampled for either type of sprouts.

Across all samples, the most abundant bacterial families were Enterobacteriaceae [30% (mean)], Bacillaceae (4.6%), and Oxalobacteraceae (4.0%). However, some families had high relative abundances on individual produce types (Fig. 2). Nearly all of the abundant bacterial families (representing ≥3% of sequences in any produce type) differed in their relative abundance among produce types. Among these families, only 2 of 19 bacterial families did not significantly differ in relative abundances across the produce types (Fig. 2). Enterobacteriaceae, for example, was the most abundant family on bean sprouts, spinach, lettuce, tomatoes, peppers, alfalfa sprouts and strawberries (at least 20%) but had substantially lower relative abundances on apples, peaches, grapes and mushrooms (Fig. 3). As previously mentioned, Enterobacteriaceae is one major group responsible for the clustering patterns described above and in Fig. 2 as those communities with high relative abundances of Enterobacteriaceae tended to cluster apart from those with lower relative abundances. Apples and peaches tended to have greater relative abundances of Microbacteriaceae and Sphingomonadaceae than other produce types. Grape surface communities displayed relatively strong contributions from the families Bacillaceae and Acetobacteraceae, and mushrooms, which showed the strongest differences from other produce types, had large relative abundances of Micrococcaceae, Sphingobacteriaceae, and Pseudomonadaceae (Fig. 2). Patterns in community composition differences at the family level were also reflected by differences in the dominant genera across the produce types. *Pantoea sp*. had a high relative abundance in most of the produce types that also had a high relative abundance of Enterobacteriaceae (those with >20% reported above). However, other produce types were generally characterized by dominant genera specific to that produce type (Table 3).

**Table 3:** Bacterial OTUs representing large proportions (>5%) of their bacterial community on a given produce type.

Produce type	OTU classification^a^	Relative abundance (%)^b^
Sprouts (bean)		
	*Pantoea sp.*	57.5
	*Klebsiella/Raoultella sp.*	14.4
Spinach		
	*Pantoea sp.*	32.4
	*Klebsiella/Raoultella sp.*	9.0
Lettuce		
	*Xanthomonas sp.*	10.0
	*Pantoea sp.*	8.9
	*Pectobacterium sp.*	8.0
	*Leuconostoc sp.*	6.9
	*Janthinobacterium sp.*	5.7
Tomato		
	*Klebsiella/Raoultella*	26.9
	*Pectobacterium sp.*	9.8
Pepper		
	*Pantoea sp.*	11.1
Sprouts (alfalfa)		
	*Acinetobacter sp.*	9.3
Strawberries		
	*Buchnera aphidicola*	23.6
	*Bacillus sp.* 1	17.1
	*Pantoea sp.*	10.4
Apple		
	*Photobacterium sp.*	5.6
Peach		
	*Microbacterium sp.*	6.2
	*Undetermined microbacteriaceae*	6.1
Grapes		
	*Bacillus sp.* 1	18.2
	*Gluconacetobacter sp*	6.0
	*Bacillus sp.* 2	5.0
Mushrooms		
	*Pseudomonas sp.*	11.3
	*Pedobacter sp.*	5.5

a Classifications determined using BLAST with the NCBI nucleotide database.

b Values represent means.

### Potential for Farming Practice to Impact Bacterial Communities

Differences in taxonomic richness on the surfaces of conventional and organic-labeled analogs depended on the produce type (Fig. 1). Organic-labeled produce had significantly greater OTU richness compared to conventional-labeled produce on spinach, lettuce, and tomatoes, and significantly lower OTU richness on peaches and grapes (*P*<0.05 for all cases, Fig. 1).

**Figure 4:**
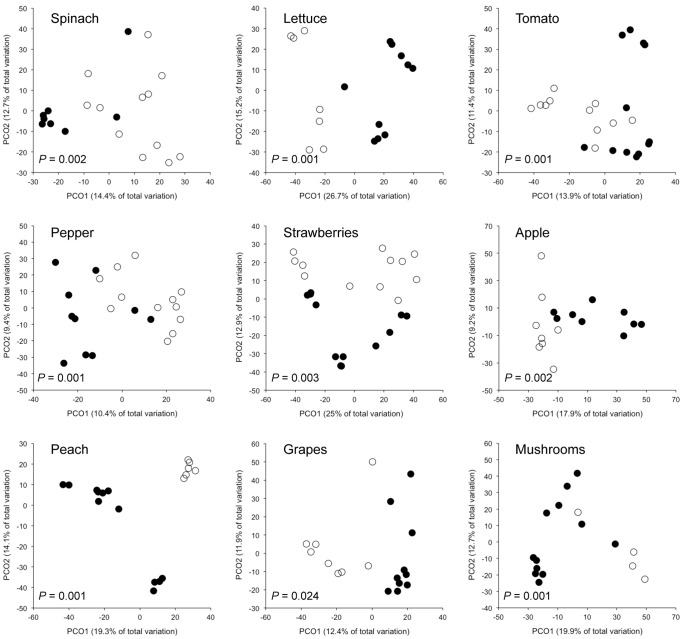
Principal coordinates analysis plots showing differences in bacterial community composition between farming practices. Plots are based on Bray-Curtis dissimilarities comparing surface bacterial communities of conventional-labeled (filled circles) and organic-labeled (open circles) produce items within each produce type. P-values were calculated using PERMANOVA.

Bacterial community composition also differed significantly between conventional and organic-labeled produce samples when taking into account variation due to produce type and store brand (*P* = 0.001), with variation in farming practice more strongly related to variation in community composition than store brand (Table 2). Furthermore, community structure differed significantly between conventional and organic-labeled produce samples within each produce type (*P*<0.05 in all cases; Fig. 4). Although the taxa driving the observed differences between conventional and organic-labeled produce were not consistent across the produce types (Table 4), conventional-labeled varieties had a greater relative abundance of Enterobacteriaceae taxa across several produce types, including spinach, lettuce, tomatoes, and peaches (Table 4). On average, enterobacteria were 64% more abundant on the surfaces of conventional labeled spinach, lettuce, tomatoes, and peaches when compared with their organic labeled equivalent (*P*<0.05 in all cases), but these differences were not evident on the surfaces of other produce types (*P*>0.05; Fig. 3). Differences among organic and conventional labeled individuals of other produce types were generally associated with families that were specific to that produce type (Table 4). For example, the communities on grapes were distinguished by a greater relative abundance of Bacillaceae on the organic-labeled grapes (Table 4).

**Table 4:** Bacterial families that differ in their relative abundances between conventional and organic-labeled equivalents within produce types.

	Taxonomy*					Relative abundance (%)	
Produce type	Phylum	Class	Order	Family	*P*	Conventional	Organic
Spinach							
	Proteobacteria	Gammaproteobacteria	Enterobacteriales	Enterobacteriaceae	0.008	71.5	48.5
	Firmicutes	Bacilli	Bacillales	Paenibacillaceae	0.028	0.3	3.6
	Firmicutes	Bacilli	Exiguobacterales	Exiguobacteraceae	0.048	2.7	5.2
Lettuce							
	Firmicutes	Bacilli	Lactobacillales	Leuconostocaceae	0.029	12.5	0
	Proteobacteria	Gammaproteobacteria	Enterobacteriales	Enterobacteriaceae	0.035	48.2	23.8
	Proteobacteria	Gammaproteobacteria	Pseudomonadales	Moraxellaceae	0.039	0.4	6.4
Tomato							
	Proteobacteria	Gammaproteobacteria	Enterobacteriales	Enterobacteriaceae	<0.001	66.8	23.8
	Actinobacteria	Actinobacteria	Actinomycetales	unclassified	0.010	0.6	3.8
	Proteobacteria	Gammaproteobacteria	Pseudomonadales	Moraxellaceae	0.015	2.9	6.8
	Proteobacteria	Alphaproteobacteria	Rhodobacterales	Rhodobacteraceae	0.015	1.1	3.1
Pepper							
	Actinobacteria	Actinobacteria	Actinomycetales	Microbacteriaceae	0.026	9.4	3.2
Apple							
	Bacteroidetes	Sphingobacteria	Sphingobacteriales	Flexibacteraceae	0.010	5.0	0.6
	Actinobacteria	Actinobacteria	Actinomycetales	Nocardioidaceae	0.036	7.1	0.6
	Proteobacteria	Alphaproteobacteria	Rhizobiales	Methylobacteriaceae	0.037	4.5	0.8
Peach							
	Actinobacteria	Actinobacteria	Actinomycetales	Microbacteriaceae	<0.001	7.4	34.5
	Proteobacteria	Gammaproteobacteria	Enterobacteriales	Enterobacteriaceae	<0.001	7.3	1.2
	Proteobacteria	Alphaproteobacteria	Rhodobacterales	Rhodobacteraceae	<0.001	1.7	4.5
	Bacteroidetes	Flavobacteria	Flavobacteriales	Flavobacteriaceae	0.012	3.4	6.4
	Proteobacteria	Betaproteobacteria	Burkholderiales	Oxalobacteraceae	0.026	6.4	0.4
	Firmicutes	Bacilli	Bacillales	Bacillaceae	0.034	0.5	3.3
Grapes							
	Firmicutes	Bacilli	Bacillales	Bacillaceae	0.007	9.6	42.0
	Firmicutes	Clostridia	Clostridiales	Clostridiaceae	0.043	3.9	1.4
	Actinobacteria	Actinobacteria	Actinomycetales	Micrococcaceae	0.049	3.0	0.9
Mushrooms							
	Proteobacteria	Alphaproteobacteria	Rhizobiales	Rhizobiaceae	0.001	4.3	0.3
	Actinobacteria	Actinobacteria	Actinomycetales	Micrococcaceae	0.003	11.7	0.4
	Bacteroidetes	Sphingobacteria	Sphingobacteriales	Sphingobacteriaceae	0.015	16.3	34
	Proteobacteria	Betaproteobacteria	Burkholderiales	Comamonadaceae	0.024	3.5	1.0

* Only families greater than or equal to 3% in one group and differed with a p-value less than 0.05 (t-test) are shown. No families met these criteria on the surfaces of strawberries.

## Discussion

Our results generally demonstrated high bacterial diversity across the eleven fruits and vegetables we analyzed. Six phylogenetically diverse phyla were well represented by the sequences in at least one produce type: Actinobacteria, Bacteroidetes, Firmicutes, Proteobacteria, and TM7 (Fig. 2). The bacterial taxa we observed were consistent with findings from other studies that have used culture-independent techniques to describe taxon abundances. We found the surface bacterial communities of spinach, lettuce, and tomatoes to be numerically dominated by Gammaproteobacteria, a pattern which has also been noted in previous studies [5], [15], [16], [37], [38]. Similarly, Ottesen et al. [18] observed that Alphaproteobacteria was the most abundant bacterial class on apples, and we found the family Sphingomonadaceae within the class Alphaproteobacteria was the most abundant family present on apples. It is more difficult to directly compare our results with the large body of research on produce-associated bacteria that has been conducted using culture-based techniques as such techniques do not typically quantify proportions of bacteria belonging to specific taxonomic groups, rather binning them into operationally-defined groups determined by the culturing media used. Furthermore, culture-based studies detect a different fraction of the bacterial community assessed using culture-independent techniques, and, in most cases, a small fraction of the total bacterial diversity [27].

We observed distinct bacterial communities and substantial variation in bacterial richness across the produce types we analyzed. The family Enterobacteriaceae, which was relatively abundant in many of the samples, contributed strongly to this variation. Enterobacteriaceae taxa dominated the community composition in the majority of produce types, but several produce types (apples, peaches, grapes, and mushrooms) harbored a very low proportion of bacteria from this family (Fig. 3). This pattern also generally coincided with patterns in richness–produce types with greater proportions of taxa belonging to Enterobacteriaceae generally had a lower taxonomic richness (Fig. 1). Other bacterial families rarely had high relative abundances on more than two produce types (Fig. 2). Taken together, these results highlight that there is minimal overlap in the dominant bacterial taxa among produce types and that there is no ‘typical’ produce-associated community. Nonetheless, one Enterobacteriaceae taxon, putatively classified as *Pantoea* sp., was particularly abundant on many of the produce types harboring large proportions of Enterobacteriaceae (Table 3). This taxon might play an important role in the ecology of their hosts as certain *Pantoea* spp. are plant pathogens [39], [40], but others may protect their hosts from disease or promote growth [19], [41]. Overall, it is not surprising there were high relative abundances of Enterobacteriaceae across many of the produce types as members of this family are known to colonize certain fruits and vegetables [42], [43]. What remains to be determined is why this family was dominant on certain produce types and relatively rare on others.

Likewise, it is difficult to unequivocally determine the specific factors responsible for driving the divergence between the bacterial communities on different produce types, but it is likely that several factors contribute to the patterns observed. Phyllosphere bacterial communities are known to strongly differ across plant species [23] likely due to variations in metabolites, physical characteristics, and symbiotic interactions with the host plant and other microbial inhabitants [37], [44]. These characteristics may similarly select for specific microbial taxa on fruits and vegetables [13], [37]. Additionally, the produce-growing medium could serve as a reservoir of bacteria that inoculate fruits and vegetables prior to harvest. However, our data do not provide evidence that this is an important mechanism for driving the relative abundances of the dominant taxa. For example, bean sprouts and spinach harbored very similar communities but the sprouts were grown hydroponically while the spinach was grown in soil (Fig. 2). Differences in handling, transport, and storage could also play a role in structuring the microbial communities [15], [16], [25]. Only the lettuce and spinach samples, for example, were rinsed prior to packaging, and storage times likely differed among the produce items. Furthermore, differences in storage temperatures among produce items due to refrigeration could influence the relative abundance of cold-tolerant bacteria [15], [16]. Additional research needs to be conducted to disentangle the contribution of these factors in structuring produce-associated bacterial communities.

In addition to variation among produce types, we also found a somewhat weaker, but significant effect, of organic versus conventional label on the produce-associated communities (Fig. 4). This effect could be attributable to a number of factors including: growing location, fertilizer use, pesticide use, other agricultural practices, and shipping and handling procedures. Likewise, some of these differences could have been due to the direct application of bacterial agents used in organic pesticides (e.g., *Bacillus* spp.) or other bacteria found in the organic manures. Nevertheless, our results suggest that differences in farming practices could be influencing the relative abundance of specific taxa on the surfaces of fresh produce available at grocery stores. Overall, Enterobacteriaceae showed consistently greater relative abundances on conventional-labeled spinach, lettuce, tomatoes, and peaches when compared to organic-labeled varieties (Table 4). Differences between the microbiota on conventional and organically farmed produce items have been reported in other studies [4], [17], [18], [26], but the differences in specific taxa may not always be consistent. For example, Oliveira et al. [4] observed a greater abundance of Enterobacteriaceae on organically farmed lettuce than its conventionally farmed equivalent via culturing techniques. Nonetheless, our data do suggest that shifts in community composition can persist for extended periods of time from the field to the grocery store and presumably, into the home of the consumer. This highlights the potential for differences in the microbiota between conventionally and organically farmed produce items to impact human health. However, as it was not our objective to differentiate between closely related taxa that may have pathogenic and non-pathogenic representatives, future research is required to assess whether the bacterial community changes associated with organic and conventional-labeled produce may impact human exposures to potential pathogens.

Our results demonstrate differences among produce types in the diversity and composition of the produce-associated bacterial communities and the potential for farming practice to affect the types of bacteria that may be consumed. Moreover, they help to establish a basis on which to pose several further questions. For example: Do the differences in communities among produce types and farming practices influence microbial degradation of produce? Do these differences infer variation in the abundance of human pathogens or human health? Do they influence taste/quality of the produce being sold? It will be important to initiate controlled experiments to determine which factors are driving the differences in bacterial communities among the different produce types and conventional and organic-labeled varieties. In particular, focused studies examining how pesticide and fertilizer use impact produce-associated microbial communities would be useful as these factors are critical in differentiating conventional and organic farming practices. There is a substantial body of literature focused on the potential effects of farming practices on food chemistry and quality with many studies finding inconsistent results [45]; this work demonstrates that the effects of different farming practices on produce-associated microbial communities can be significant and are clearly worthy of further investigation.

## Supporting Information

Figure S1 **Principal coordinate analysis plot showing bacterial community composition by produce type.** This plot is based on Bray-Curtis dissimilarities of samples rarefied at 200 sequences per sample.
(TIF)
