# Supplementary figures and images for: Bacterial Communities Associated with the Surfaces of Fresh Fruits and Vegetables
Source: PLoS One. 2013 Mar 27;8(3):e59310. doi: 10.1371/journal.pone.0059310 (PMC3609859; doi:10.1371/journal.pone.0059310)

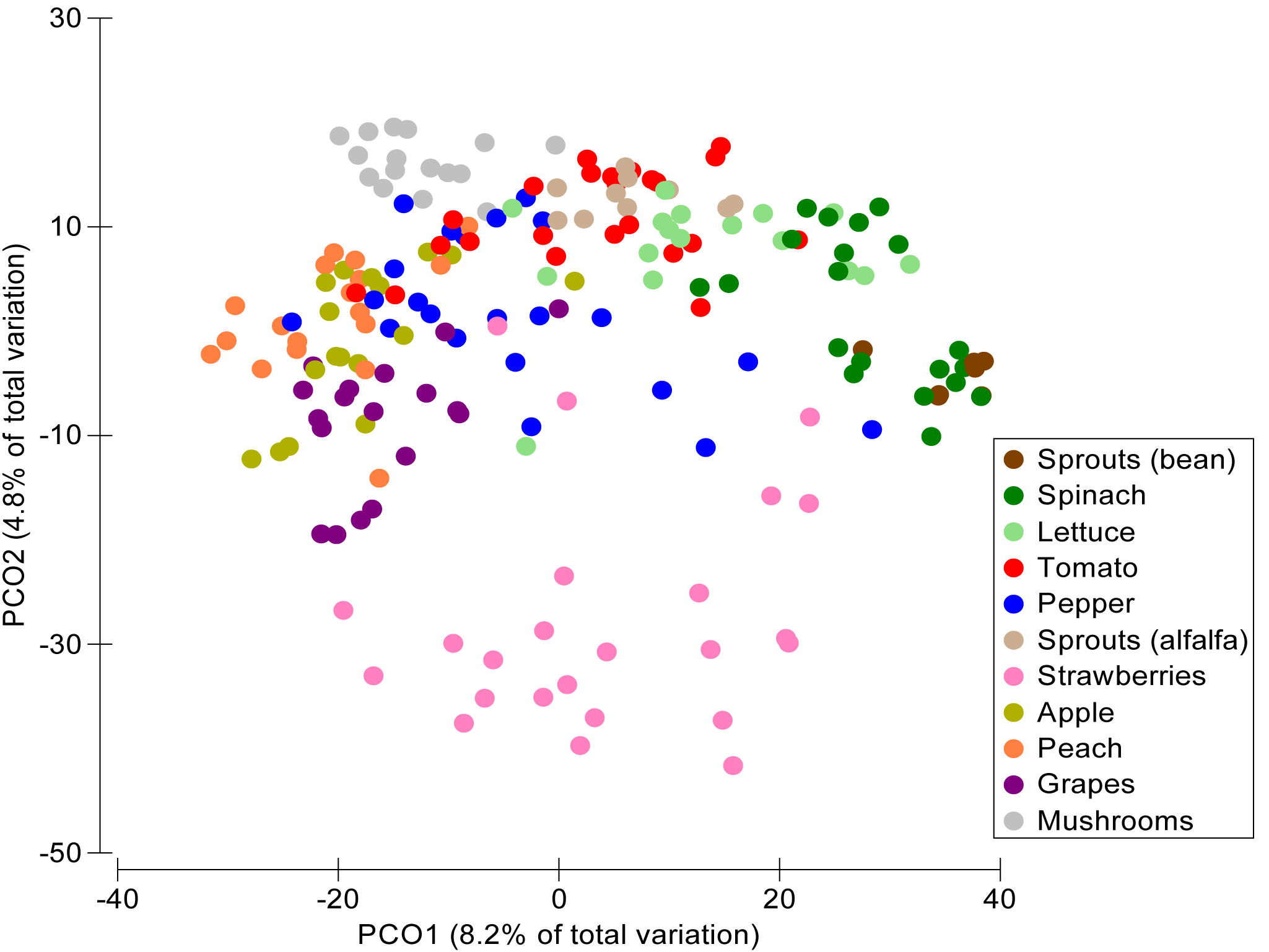

Supplement: Figure S1 — Principal coordinate analysis plot showing bacterial community composition by produce type. This plot is based on Bray-Curtis dissimilarities of samples rarefied at 200 sequences per sample. (TIF) [file pone.0059310.s001.tif]
